# Overweight/obesity in young adulthood interacts with aspects of EBV infection in MS etiology

**DOI:** 10.1212/NXI.0000000000000912

**Published:** 2020-12-15

**Authors:** Anna Karin Hedström, Nicole Brenner, Julia Butt, Jan Hillert, Tim Waterboer, Tomas Olsson, Lars Alfredsson

**Affiliations:** From the Department of Clinical Neuroscience (A.K.H., J.H., T.O., L.A.), Karolinska Institutet, Stockholm, Sweden; Infections and Cancer Epidemiology (N.B., J.B., T.W.), German Cancer Research Center (DKFZ), Heidelberg; Center for Molecular Medicine (J.H., T.O.), Karolinska Institutet at Karolinska University Hospital, Solna, Sweden; and Institute of Environmental Medicine (L.A.), Karolinska Institutet, Stockholm, Sweden.

## Abstract

**Objective:**

Because obesity affects the cellular immune response to infections, we aimed to investigate whether high body mass index (BMI) in young adulthood and high Epstein-Barr nuclear antigen 1 (EBNA-1) antibody levels interact with regard to MS risk. We also aimed at exploring potential 3-way interactions between BMI at age 20 years, aspects of Epstein-Barr virus (EBV) infection (high EBNA-1 antibody levels and infectious mononucleosis [IM] history, respectively) and the human leukocyte antigen (HLA)-DRB1*15:01 allele.

**Methods:**

Using Swedish population-based case-control studies (5,460 cases and 7,275 controls), we assessed MS risk in relation to interactions between overweight/obesity at age 20 years, IM history, EBNA-1 levels, and HLA-DRB1*15:01 status by calculating ORs with 95% CIs using logistic regression. Potential interactions were evaluated on the additive scale.

**Results:**

Overweight/obesity, compared with normal weight, interacted significantly with high (>50th percentile) EBNA-1 antibody levels (attributable proportion due to interaction 0.2, 95% CI 0.1–0.4). The strength of the interaction increased with higher category of EBNA-1 antibody levels. Furthermore, 3-way interactions were present between HLA-DRB1*15:01, overweight/obesity at age 20 years, and each aspect of EBV infection.

**Conclusions:**

With regard to MS risk, overweight/obesity in young adulthood acts synergistically with both aspects of EBV infection, predominantly among those with a genetic susceptibility to the disease. The obese state both induces a chronic immune-mediated inflammation and affects the cellular immune response to infections, which may contribute to explain our findings.

MS is a multifocal inflammatory disease of the CNS with underlying genetic and environmental factors. Variations in the human leukocyte antigen (HLA) region influence susceptibility to MS with the main effect originating from the Class II *DRB1* gene.^1^ Environmental and lifestyle factors increasing MS risk include smoking,^2^ low sun exposure,^3^ low vitamin D status,^4^ high body mass index (BMI),^5,6^ and Epstein-Barr virus (EBV) infection.^7^ High BMI during adolescence and young adulthood, but not at the time of MS onset, has repeatedly been associated with an increased risk of developing MS.^5,6^ The risk increases in a dose-dependent manner.^6^

Elevated Epstein-Barr nuclear antigen 1 (EBNA-1) antibody levels and infectious mononucleosis (IM) history are aspects of EBV infection that seem to represent separate risk factors for MS.^7^ An additive interaction has been observed between high BMI at age 20 years and a history of IM,^8^ but whether a similar interaction occurs between obesity and elevated EBNA-1 antibody levels is unclear.

Because most environmental and lifestyle risk factors for MS seem to act synergistically with the main genetic risk factor for MS, the HLA-DRB1*15:01 allele,^7,9^ it is also of interest to study both separate and joint effects of these factors.

Using Swedish population-based case-control studies, comprising 5,460 cases and 7,275 controls, we thus aimed to investigate potential 2- and 3-way interactions between BMI at age 20 years, aspects of EBV infection, and presence of DRB1*15:01.

## Methods

### Study design and study subjects

We used 2 Swedish population-based case-control studies on environmental and genetic risk factors for MS: Epidemiological Investigation of Multiple Sclerosis (EIMS) and Genes and Environment in Multiple Sclerosis (GEMS). The study population comprised the general population aged 16–70 years. In EIMS, newly diagnosed cases of MS were recruited from neurology clinics, including all university hospitals in Sweden. Cases were diagnosed by a neurologist according to the 2005 or 2010 McDonald criteria.^10,11^ For each case, we randomly selected 2 controls from the national population register, frequency matched by age in 5-year age strata, sex, and residential area. The participants were included between April 2005 and June 2015.

In GEMS, we identified prevalent cases of MS from the Swedish National MS Registry.^12^ All cases in both studies fulfilled the 2005 or 2010 McDonald criteria.^10,11^ One control for each case was selected in the same manner as in EIMS. All participants were recruited between November 2009 and November 2011.

To increase the number of controls, we also used controls from the Epidemiological Investigation of Rheumatoid Arthritis (EIRA), which is a population-based case-control study with incident cases of rheumatoid arthritis, designed in the same manner as EIMS, using a similar study population in southern and middle parts of Sweden. The EIRA controls were not matched on age, sex, or residential area to MS cases in either EIMS or GEMS. The study has been described in detail elsewhere.^13^

### Data collection

We used a standardized questionnaire to collect information regarding environmental exposures and lifestyle factors. The response rate was 93% for cases and 73% for controls in EIMS, 82% for cases and 66% for controls in GEMS, and 75% for controls in EIRA.

In all studies, participants were asked whether they had ever had IM, and if yes, at what age the infection occurred. Those who were unsure regarding IM history were excluded. For each case in EIMS and GEMS, the clinical onset of disease was defined as the index year. The corresponding controls were given the same index year. IM history was considered before the index year and was recorded as either reported infection or no infection.

Self-reported information was obtained regarding current body height and body weight at age 20 years. Subjects younger than 20 years at the index year were excluded. Using current height, we calculated BMI at age 20 years by dividing weight in kilograms by height in meters squared.

All participants were asked to provide a blood sample and those who did not donate blood were excluded from the main analysis. The number of study subjects in each study is presented in table 1.

**Table 1:**
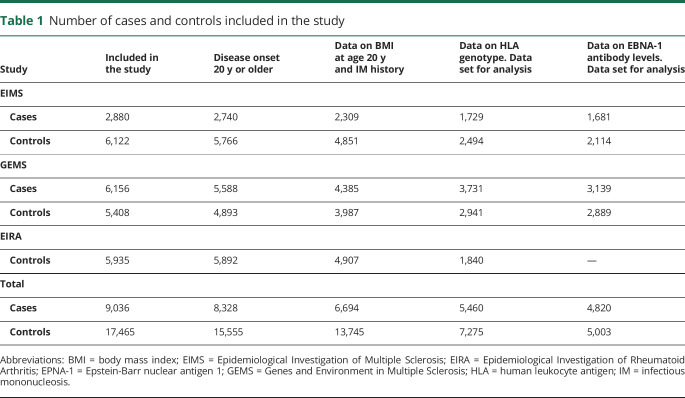
Number of cases and controls included in the study

### Potential confounding variables

Ancestry was dichotomized into Swedish or non-Swedish. Educational level was categorized into no postsecondary education, postsecondary education, or university degree. Based on 3 questions about exposure to ultraviolet radiation where each answer alternative was given a number ranging from 1 (the lowest exposure) to 4 (the highest exposure), we constructed an index by adding the numbers together and thus acquired a value between 3 and 12.^14^ Sun exposure was then dichotomized based on the 25th percentile among controls. Smokers were dichotomized into ever or never smokers. Exposure to passive smoking at home or at work on a daily basis was dichotomized into ever or never exposed. Alcohol consumption was categorized into the following subgroups based on the amount of alcohol intake per week: low consumption (<50 g/wk for women and <100 g/wk for men), moderate consumption (50–112 g/wk for women and 100–168 g/wk for men), and high consumption (>112 g/wk for women and >168 g/wk for men). The cutoffs were the same as those used by Statistics Sweden, a government agency that produces official statistics.

### Standard protocol approvals, registrations, and patient consents

Ethical approval for both EIMS and GEMS was obtained from the Regional Ethical Review Board at Karolinska Institutet. All participants gave their written informed consent.

### Genotyping and measurement of EBNA-1 antibody levels

In EIMS and GEMS, *HLA-DRB1* and *HLA-A* alleles were determined at 4-digit resolution. Genotyping was performed on the MS replication chip,^15^ which is based on an Illumina exome chip, and HLA was then imputed with HLA*IMP:02.^16^ In EIRA, *HLA-DRB1* genotypes were obtained as previously described.^13^ In EIMS and GEMS, we used multiplex serology to detect immunoglobulin G antibodies against the EBNA-1 peptide segment (aa 385–420),^17,18^ which has been identified as the primary EBNA-1 fragment associated with MS risk.^19^ Dual-laser flow-based detection was used to quantify the antibodies in median flourescence intensity (MFI) units. We dichotomized EBNA-1 antibody levels based on the median seroreactivity among controls (5,620 MFI) into groups with high and low EBNA-1 antibody levels.^7^ To study whether the potential interaction between obesity at age 20 years and high EBNA-1 antibody levels was affected by increasing EBNA-1 levels, we also divided the participants into 4 groups based on the seroreactivity among controls at the 50th, 75th, and 95th percentiles.

### Statistical analysis

All analyses were adjusted for study, age, sex, residential area, ancestry, smoking, and when appropriate for A*02:01, DRB1*15:01, and IM history. Adjustments were also made for educational level, sun exposure habits, passive smoking, and alcohol consumption, but these factors had minor influence on the results and were not retained in the final analyses.

Subjects characterized by BMI at age 20 years, EBNA-1 and IM status were compared with regard to MS risk, by calculating ORs with 95% CIs using unconditional logistic regression models.^20^ Potential interactions were analyzed using departure from additivity of effects as criterion of interaction. Synergistic effects between overweight/obesity at age 20 years and each aspect of EBV infection were evaluated by calculating the attributable proportion due to interaction and relative excess risk due to interaction together with 95% CI.^21,22^ Superadditive associations between overweight/obesity at age 20 years, HLA-DRB1*15, and a history of IM (or high EBNA-1 antibody levels) were calculated by comparing the joint effect of the 3 risk factors to the situation when each one acts separately, using the total relative excess risk due to interaction.^18^

As sensitivity analyses, we performed the interaction analyses stratified by study. We also conducted all analyses restricted to include subjects with data on HLA alleles as well as anti–EBNA-1 antibody levels. These analyses were further adjusted for DRB1*15:01 when appropriate and for the following alleles from other HLA regions that have been shown to influence MS risk independently of DRB1*15:01 status^1^: DRB1*03:01, DRB1*13:03, DRB1*08:01, B*44:02, B38:01, B44:02, DQA1*01:01, DQB1*03:02, and DQBI*03:01.^1^ Homozygote correction was made for DRB1*15:01, DRB1*03:01, and A*02:01. All analyses were conducted using Statistical Analysis System version 9.4.

### Data availability

Anonymized data will be shared by request from any qualified investigator who wants to analyze questions that are related to the published article.

## Results

Among the cases, the mean age at disease onset was 33.7 (SD 10.4) years. The majority of cases in EIMS were recruited within 1 year after the diagnosis, and the questionnaires were completed after a median of 2.0 years following MS onset. In GEMS, the median duration from disease onset to study inclusion was 18.0 (SD 11.7) years. Selected characteristics of cases and controls are presented in table 2. Characteristics were similar between those who donated blood and those who did not (table e-1, links.lww.com/NXI/A374).

**Table 2:**
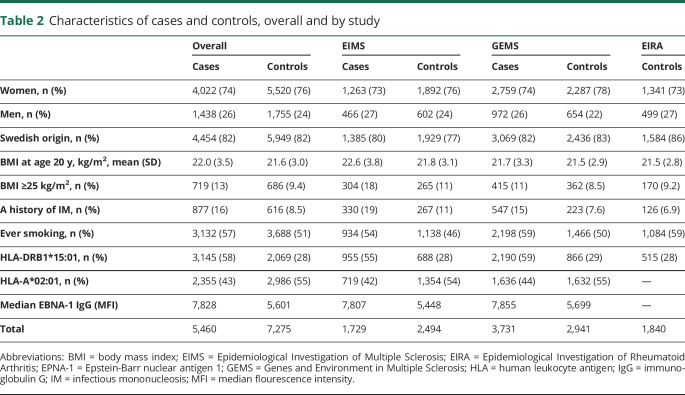
Characteristics of cases and controls, overall and by study

### BMI at age 20 years and a history of IM

Overweight/obesity at age 20 years and a history of IM synergistically increased the risk of MS. Overall, IM history among nonoverweight subjects was associated with a 90% increased risk of MS, whereas overweight/obesity at age 20 years (BMI ≥25 kg/m^2^) with no history of IM was associated with a 40% increased MS risk. Compared with nonoverweight subjects without IM history, the combination of the 2 risk factors increased MS risk 5-fold.

### BMI at age 20 years and EBNA-1 status

A similar but less pronounced interaction was observed between BMI at age 20 years and high EBNA-1 antibody levels. The interaction became stronger with increasing category of EBNA-1 antibody levels, using a variety of different cut-points (table 3). Our findings remained significant when subjects with a history of IM were excluded.

**Table 3:**
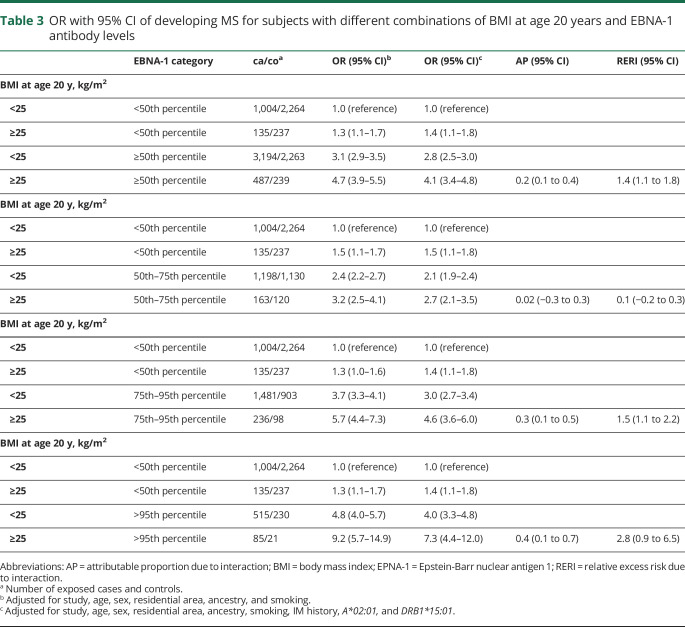
OR with 95% CI of developing MS for subjects with different combinations of BMI at age 20 years and EBNA-1 antibody levels

### DRB1*15 status, BMI at age 20 years, and aspects of EBV infection

With regard to MS risk, 2-way interactions were present between DRB1*15:01 and overweight/obesity at age 20 years, between DRB1*15:01 and each aspect of EBV infection, and between overweight/obesity at age 20 years and each aspect of EBV infection (tables 4 and 5).

**Table 4:**
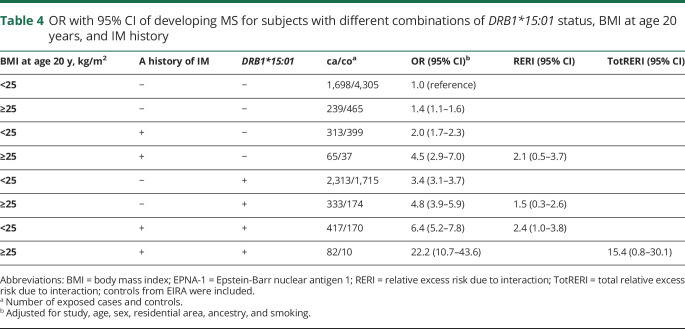
OR with 95% CI of developing MS for subjects with different combinations of DRB1*15:01 status, BMI at age 20 years, and IM history

**Table 5:**
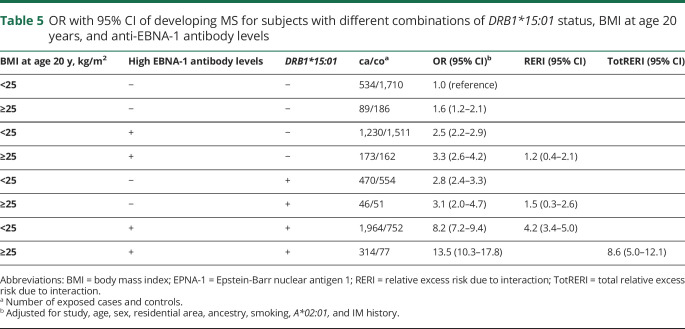
OR with 95% CI of developing MS for subjects with different combinations of DRB1*15:01 status, BMI at age 20 years, and anti-EBNA-1 antibody levels

Furthermore, significant 3-way interactions on the additive scale were observed between DRB1*15:01, BMI at age 20 years, and each aspect of EBV infection (IM history and high EBNA-1 antibody levels, respectively) (tables 4 and 5). The results presented in tables 4 and 5 are illustrated in the figure.

**Figure:**
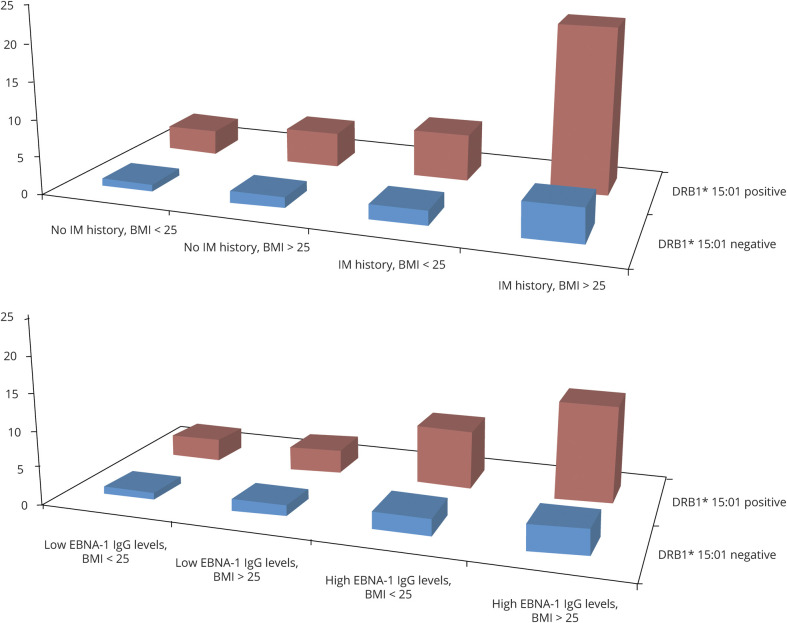
OR of developing MS for subjects with different combinations of DRB1*15:01, overweight/obesity (>25 kg/m^2^), and IM history and EBNA-1 status, respectively, compared with unexposed DRB1*15:01-negative subjects Based on data from tables 4 and 5.

All main findings remained significant when the analyses were stratified by study (EIMS and GEMS). The overall interaction between BMI at age 20 years and high EBNA-1 antibody levels, stratified by study, is presented in table e-2, links.lww.com/NXI/A374. All findings also remained significant when we performed the analyses restricted to subjects with complete data on HLA alleles and EBNA-1 antibody levels (data not shown).

## Discussion

According to our findings, overweight/obesity in young adulthood acts synergistically with both a history of IM and high EBNA-1 antibody levels. The effect estimates for the interaction between BMI at age 20 years and EBNA-1 antibody levels became stronger with increasing category of antibody levels. Furthermore, significant 3-way interactions on the additive scale were observed between DRB1*15:01, BMI at age 20 years, and each aspect of EBV infection.

Adipose tissue is considered an active endocrine organ that results in a strong proinflammatory environment in subjects with obesity. Inflammatory mediators such as interleukin 6, C-reactive protein, and tumor necrosis factor alpha significantly correlate with BMI. Obesity has been associated with a decreased number of regulatory T and B subsets, expansion of Th17 cells, and promotion of autoantibodies, which may lead to breakdown of self-tolerance and promote the onset and progression of autoimmune responses.^23–25^ Obesity contributes to the development of a number of inflammatory and autoimmune diseases, including MS.^26,27^ Obesity also results in a state of immunodeficiency, including altered lymphocyte functionality, rendering obese people more susceptible to infections.^28^ Immunodeficiency due to obesity may thus alter immune responses to pathogens and increase the risk of an inflammatory response directed at self-antigens. Several environmental factors that predispose to MS, including both high BMI and EBV infection, seem to act long before the clinical onset of the disease. This suggests a sensitive period for lifestyle and environmental factors in MS or may reflect the existence of a prodromal phase for the disease.

Both EIMS and GEMS, as well as EIRA, retrospectively gathered information regarding environmental and lifestyle factors. EIMS included incident cases to minimize recall bias, whereas GEMS was based on prevalent MS cases and recall bias may therefore be more substantial in this study. However, a validation study among women in the Nurses' Health Study found high correlations between recalled and measured past weight.^29^

There is a risk of misclassification when dichotomizing subjects into those with and without self-reported IM history. Because infection by other pathogens than EBV may cause an IM-like illness, subjects may unknowingly have had IM. However, this misclassification is not expected to differ between cases and controls. Furthermore, the risk of MS among subjects with a history of IM was in accordance with that of previous studies on past IM and MS risk.^30^ EBNA-1 antibody levels after MS onset were assumed to reflect levels before disease onset. This assumption is supported by findings that EBNA-1 antibody levels become elevated between 15 and 20 years before the clinical onset of MS and thereafter remain constant over time.^31^ Another concern is that the recruitment of cases and controls may introduce selection bias.

Because the Swedish health care system provides equal free of charge access to medical services for all Swedish citizens, most MS cases are referred to neurologic units. In both MS studies, selection bias was minimized by the population-based design. Although there was proportion of nonresponders among the controls, this bias is probably modest because the prevalence of lifestyle factors, such as smoking and socioeconomic status, among controls was consistent with that of the general population.^32^ Subjects who were excluded due to unknown IM history did not differ with regard to obesity at age 20 years, EBNA-1 antibody levels, or DRB1*15:01 status compared with those with known IM history. Furthermore, there were no significant differences with respect to age, sex, BMI at age 20 years, or IM history between those donated blood and those who did not, indicating that selection bias did not take place in this step.

The worldwide prevalence of overweight/obesity during childhood and young adulthood has increased over the last decades and has emerged as a serious public health concern.^33^ The increasing MS incidence in some countries may, at least to some extent, be explained by the increasing prevalence of obesity. Overweight and obesity are largely preventable, and intervention efforts need to be high priority to reduce long-term health consequences, including inflammatory diseases such as MS.

In summary, overweight/obesity in young adulthood acts synergistically with both aspects of EBV infection, predominantly among those with a genetic susceptibility to the disease. The obese state both induces a chronic immune-mediated inflammation and affects the cellular immune response to infections, which may contribute to explain our findings. Our data reinforce the importance of intervention efforts against childhood and adolescent obesity to reduce MS incidence.

## Glossary

BMI: body mass index
EBV: Epstein-Barr virus
EIMS: Epidemiological Investigation of Multiple Sclerosis
EIRA: Epidemiological Investigation of Rheumatoid Arthritis
EPNA-1: Epstein-Barr nuclear antigen 1
GEMS: Genes and Environment in Multiple Sclerosis
HLA: human leukocyte antigen
IM: infectious mononucleosis
MFI: median flourescence intensity
